# Re-evaluating treatment strategies: a closer look at the FOLFOX-nab-Paclitaxel approach in the immunotherapy era for metastatic gastric cancer

**DOI:** 10.1093/oncolo/oyag120

**Published:** 2026-04-03

**Authors:** Aysegul Merc Cetınkaya, Yusuf Ilhan

**Affiliations:** Department of Medical Oncology, Akdeniz University Faculty of Medicine Hospital, Pinarbasi Neighborhood Dumlupinar Boulevard, Antalya, 07070, Turkey; Department of Medical Oncology, Antalya City Hospital, Gocerler Neighborhood, 5379 Street, Antalya, 07080, Turkey

To the Editor

We read with great interest the article by Drayer et al. “A phase II study of FOLFOX combined with nab-paclitaxel in the treatment of metastatic or advanced unresectable gastric, gastroesophageal junction adenocarcinoma: a Big Ten Cancer Research Consortium trial”^1^ published in your journal. We would like to thank the authors for this important study on metastatic gastric cancer, which continues to have a low survival rate. However, we have some concerns about the study and wish to provide comments that may be useful for future research.

Firstly, patients were included in the study between 2017 and 2023. At the outset, the standard first-line treatment for this patient group was the FOLFOX regimen. However, following 2021, significant studies emerged emphasizing the importance of immunotherapy in the treatment of metastatic gastric cancer, leading to changes in clinical guidelines.^2–4^ Currently, it is recommended that patients with PD-L1 CPS >5 and >10 receive FOLFOX in combination with Nivolumab and Pembrolizumab. In your study, PD-L1 levels were found to be greater than 1% in 67% of the patients. If there is no contraindication to receiving immunotherapy, it would be more appropriate to reassess these patients’ PD-L1 levels and consider a chemo-immunotherapy combination if the score exceeds 5%. Continuing the patient enrollment under the same conditions after these developments could be considered an ethical violation.

Secondly, the study mentions that the investigational regimen was not inferior to the standard treatment and that no unexpected or concerning adverse effects were observed. However, the FOLFOX regimen used in the study does not include a 5-FU bolus, as in the standard regimen. We are concerned that this difference may have contributed to changes in the observed adverse effects.

Lastly, in the conclusion section, the study regimen is compared with the FLOT regimen. However, it is important to note that the FLOT regimen is typically used for the perioperative treatment of locally advanced gastric cancer and is not preferred in the metastatic setting. Furthermore, its toxicity is known to be greater than that of FOLFOX. Therefore, we do not believe that comparing the study regimen with FLOT and drawing conclusions in this context constitutes a valid assessment.

We would like to reiterate our gratitude to the authors for their efforts in seeking better survival outcomes without immunotherapy in an era where immunotherapy has become paramount. Their search for alternative treatment regimens is truly commendable, and the results obtained are undeniably significant. Although improvements in survival rates have been observed with the introduction of immunotherapy, there remains much work to be done in the treatment of metastatic gastric cancer. Additionally, unfortunately, some patients may not be eligible for immunotherapy. In light of our critiques, future studies involving larger, more homogeneous, and appropriately selected patient groups could yield more accurate results for metastatic gastric cancer cases that should receive non-immunotherapy treatments, potentially providing significant insights that could change clinical practice.

Sincerely
